# Tissue Kallikrein Activity, Detected by a Novel Method, May Be a Predictor of Recurrent Stroke: A Case-Control Study

**DOI:** 10.1155/2015/159750

**Published:** 2015-09-14

**Authors:** Xiao Ran, Qin Zhang, Dao Wen Wang

**Affiliations:** ^1^Department of Emergency Medicine, Tongji Hospital, Tongji Medical College, Huazhong University of Science and Technology, Jiefang Avenue No. 1095, Wuhan 430030, China; ^2^Department of Anesthesia, Tongji Hospital, Tongji Medical College, Huazhong University of Science and Technology, Jiefang Avenue No. 1095, Wuhan 430030, China; ^3^The Institute of Hypertension and Department of Internal Medicine, Tongji Hospital, Tongji Medical College, Huazhong University of Science and Technology, Jiefang Avenue No. 1095, Wuhan 430030, China

## Abstract

*Aim*. Tissue kallikrein (TK) protein content in plasma has been shown to be negatively associated with both incident and recurrent strokes. The aims of this study were to develop a novel method for detecting TK activity and to investigate its association with event-free survival over 5 years in Chinese first-ever stroke patients. *Methods*. We designed a case-control study with 321 stroke patients (174: ischemic stroke, 147: hemorrhagic stroke) and 323 healthy local controls. TK activity was measured by a novel assay utilizing the immunological characteristics of TK and the catalysis of benzoyl arginine ethyl ester hydrochloride (BAEE). *Results*. TK protein levels above 0.200 mg/L in plasma were not associated with urinary TK activity or the risk of stroke recurrence. TK activity was significantly lower in stroke patients compared with controls (1.583 ± 0.673 Eu/mL versus 1.934 ± 0.284 Eu/mL, *P* < 0.001). After adjusting for traditional risk factors, TK activity was negatively associated, in a dose-response manner, with the risk of overall stroke recurrence and positively associated with event-free survival during a 5-year follow-up (relative risk (RR), 0.69; 95% CI, 0.57–0.84; *P* < 0.001). *Conclusions*. Our findings suggest that urinary TK activity may be a stronger predictor of stroke recurrence than plasma TK levels.

## 1. Introduction

Stroke has become one of the leading causes of death and long-term disability in many countries and at all levels of economic development [1], with a high occurrence and recurrence rate [2, 3]. In China, 2.5 million people have stroke and more than 1 million die from stroke-related causes every year. The total cost of hospitalization for patients with stroke was twice as much as per capita annual income of China and still growing [4]. Conventional cardiovascular risk factors, such as abnormal lipid metabolism, smoking, diabetes, and hypertension, account for a considerable portion of stroke events [5]. However, most subjects exhibiting these risk factors do not develop stroke during their lifetime, suggesting that additional factors are involved.

The tissue kallikrein- (TK-) kinin/bradykinin system is recognized as an important modulator of the cardiovascular, renal, and central nervous systems [6]. TK, a major member of the ubiquitously expressed kallikrein family, releases vasoactive bradykinin and kinin peptides from low molecular weight kininogen [7]. Kinins exert their action through two G-protein-coupled receptors, kinin B1 and kinin B2 receptors in the endothelium [8], which mediate regulation of blood pressure, smooth muscle contraction and relaxation, vascular cell growth, vascular permeability, inflammatory cascades, electrolyte balance, and pain induction [9, 10]. The role of TK was studied in numerous experimental studies focusing on this disease using strategies like kallikrein gene transfer, tissue kallikrein infusion, and/or human kallikrein over expressing animals [11]. These basic studies have demonstrated that TK can reduce elevated blood pressure and inhibit remodeling in the heart and arteries [12, 13]; attenuate ischemic stroke in an animal model [14]; reduce the rate of smooth muscle cell proliferation [15], atherosclerosis, and apoptosis [16]; protect against ischemia/reperfusion damage [17]; and enhance angiogenesis [18].

Early clinical studies have indicated an inverse relationship between urinary TK activity and blood pressure in patients with hypertension [19, 20]. Our previous study investigated the association of TK protein content in plasma with stroke and coronary artery disease (CAD) [21, 22]. In a multicenter case-control study conducted in China, we found that the plasma TK level was negatively associated with incident and recurrence of stroke. However, the relationship between plasma TK level and stroke recurrence disappeared when the plasma TK level exceeded 0.200 mg/L. We also found that the elevated TK levels are positively associated with the presence of CAD and negatively associated with the severity of CAD and that the TK level might be a strong and independent biomarker for stroke. However, no study to date has investigated the relationship between TK activity and stroke. We therefore developed a novel method for determining TK activity in urine and assessed its association with stroke and the long-term recurrence of cerebrovascular and cardiovascular events in a 5-year follow-up study of Chinese patients with first-ever stroke.

## 2. Methods

### 2.1. Study Population and Data Collection

The study population has been described in detail in a previous publication [21]. Experiments were conducted in accordance with the principles of Declaration of Helsinki. The study protocol was approved by the Ethics Committee of Tongji Hospital, and informed written consent was obtained from all participants. We recruited participants from November 2000 to November 2001 from 5 hospitals in Wuhan, China. Stroke patients, described previously, included cerebral infarction (atherothrombosis and lacunar infarction) and cerebral hemorrhage. Other types of stroke (transient ischemic attack, subarachnoid hemorrhage, embolic brain infarction, brain tumors, cerebrovascular malformation, cardioembolic stroke, and documented atrial fibrillation); severe systemic disease; inflammatory and autoimmune diseases; and serious chronic diseases were excluded from the study. Confirmation of stroke was based on strict neurological examination, computed tomography (CT), or magnetic resonance imaging (MRI) according to the International Classification of Diseases, Ninth Revision as described previously [21]. Ethnically matched controls were randomly selected from inpatients with minor illness who were free of neurological diseases. In total, 321 patients (174: ischemic stroke, 147: hemorrhagic stroke) and 323 control subjects were recruited. All participants underwent a standardized interview, and data on traditional stroke risk factors were recorded for all subjects. These included gender, age, body mass index (BMI), systolic blood pressure (SBP), diastolic blood pressure (DBP), triglyceride (TG), total cholesterol (TC), high-density lipoprotein (HDL), cholesterol, history of diabetes mellitus, hypertension, hyperlipidemia, and smoking habits. These criteria were described in a previous study [21].

To evaluate the association of TK activity with long-term clinical outcomes in stroke patients, follow-up visits of patients for a median of 5 years (range: 4–5.5 years) were performed by telephone or household contact after the initial stroke. The primary endpoint was recurrence of stroke. The onset of coronary heart disease or death was secondary endpoints.

### 2.2. TK Activity Assays

**Figure 1:**
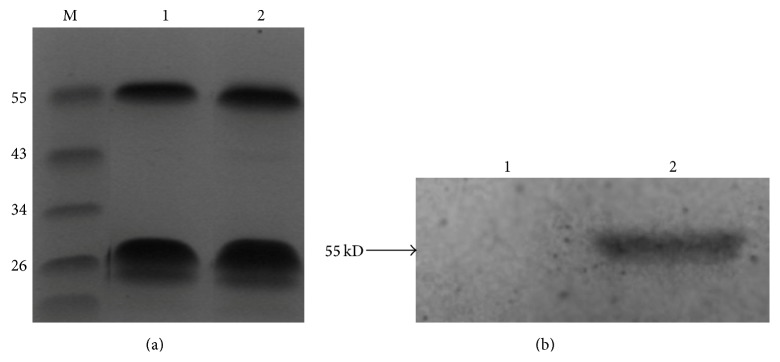
SDS-PAGE and Western blotting with polyclonal and monoclonal antibodies. (a) the purified polyclonal antibody (lanes 1 and 2) was separated using SDS-PAGE with the molecular mass markers indicated in lane M. (b) PEGX-4T1 control protein (lane 1) and GST-TK fusion protein (lane 2) were transferred onto nitrocellulose membranes for Western blotting using a polyclonal antibody.

Plasma and urinary samples were collected after an overnight fast and stored at −80°C. Specimens collected from stroke patients were taken after the acute phase of the initial stroke (between 1 month and 1 year after the onset). TK activity was measured using a novel assay utilizing TK immunological characteristics and the catalysis of benzoyl arginine ethyl ester hydrochloride (BAEE). To generate a polyclonal antibody specific for TK, a synthetic TK peptide consisting of 11 amino acids (AC-Glu-Asn-His-Thr-Arg-Gln-Ala-Asp-Glu-Asp-Thr-COOH) was fused to keyhole limpet hemocyanin (KLH) and used to immunize rabbits. The antibody was purified by affinity column chromatography. SDS-PAGE (12%) and Western blot were used to visualize the specificity of the purified antibody. The polyclonal antibody was pure (Figure 1) and had a high titer (1 : 25600 dilution). The antibody specific for TK was precoated onto microplates and incubated for 24 hours at 4°C. After aspirating each well and washing, 1% BSA-PBS was used as a blocking buffer for 1 hour at 37°C. All samples consisted of 10 *μ*L of urine added to 90 *μ*L of PBS. Samples were then added to the appropriate microplate wells and incubated for 1 hour at 37°C. After aspirating each well and washing, TK-catalyzed BAEE and the remaining BAEE were reacted with hydroxylamine and ferric chloride. TK activity was then detected by its esterolytic activity on synthetic ester substrates [23–26].

### 2.3. Statistical Analysis

Statistical and association analyses were performed using SPSS 18.0 software (SPSS Inc., Chicago, IL, USA). Continuous variables were expressed as mean ± standard deviation and statistical significance was tested using the independent samples *t*-test or a 1-way analysis of variance. Categorical variables were analyzed using the chi-square statistic test. Bivariate analyses were conducted to assess the relationship between plasma TK level and urinary TK activity. Pearson's and Spearman's correlation coefficient *r* was calculated to test for associations between both continuous and categorical variables. The relationships of TK activity with traditional cardiovascular risk factors of stroke patients were tested in multivariable forward stepwise linear regression analysis. The distribution of continuous variables was tested based on skewedness and kurtosis. Because TK activity had a skewed distribution, for further analysis the TK activity levels in stroke patients and controls were categorized by 4 quartiles. TK activity was then used as a continuous, categorical variable in the subsequent analysis. Correlations between TK activity and other risk factors were also calculated. Unconditional logistic regression analysis was run for stroke and separately for ischemic and hemorrhagic stroke to estimate odds ratio (OR), together with 95% confidence intervals (CIs). Multivariate Cox proportional hazard models were then performed to estimate relative risks (RRs) and 95% CIs for the effect of TK activity on independent risk of recurrent stroke. Unadjusted models and models adjusted for traditional cardiovascular risk factors (described above) were calculated. Kaplan-Meier survival estimates were used to examine the association between TK activity, stroke recurrence, and event-free survival time. A *P* value of less than 0.05 was considered as statistically significant and all probabilities were two-tailed.

## 3. Results

### 3.1. Descriptive Statistics of TK Activity in Urine

**Figure 2:**
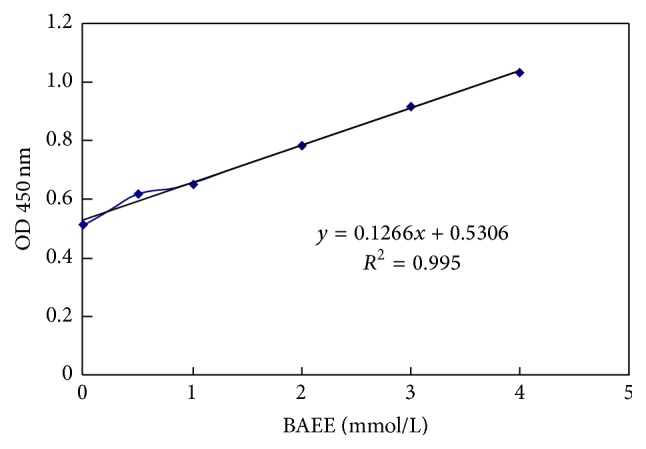
Standard curve of TK activity measurement.

Results generated using TK activity assays indicated that TK activity is linear (*r* = 0.995, Figure 2). The range of detection of the assay was 1.27 Eu–0.040 Eu. Of the 20 people for whom 3 TK activity measurements during a 6-month period were obtained, the intraindividual coefficient of variation was 6.9%, reflecting the long-term stability of TK activity. The assay remained stable, as indicated by an intraindividual coefficient of variation of 4.7% for 15 samples over 5 freeze-thaw cycles.

The distribution of TK activity was positively skewed (skewedness = −0.602, standard error = 0.096; kurtosis = 1.879, standard error = 0.192). There was a significant correlation between plasma TK levels and urinary TK activity when the plasma TK level was below 0.200 mg/L (*r* = −0.100; *P* = 0.016). This result is similar to our other study, in which we found that plasma TK levels below 0.200 mg/L were negatively associated with recurrent stroke [21]. However, this association disappeared when the plasma TK level was over 0.200 mg/L (*r* = 0.150; *P* = 0.244).

### 3.2. Study Population Characteristics

A total of 321 stroke patients (174: ischemic stroke, 147: hemorrhagic stroke) and 323 local control subjects were enrolled in the study. The clinical and laboratory characteristics of the participants at baseline are presented in Table 1. Stoke patients had a significantly higher proportion of hypertension (*P* = 0.034), diabetes (*P* = 0.016), cigarette smoking habits (*P* < 0.001), increased body weight (*P* = 0.022), higher levels of triglycerides (*P* < 0.001), lower levels of HDL cholesterol (*P* < 0.001), and total cholesterol (*P* < 0.001). There were no statistically significant group differences in terms of gender, age, SBP, and BMI. In particular, TK activity was significantly lower in total stroke patients than in controls (1.583 ± 0.673 versus 1.934 ± 0.284 Eu/mL, *P* < 0.001).

**Figure 3:**
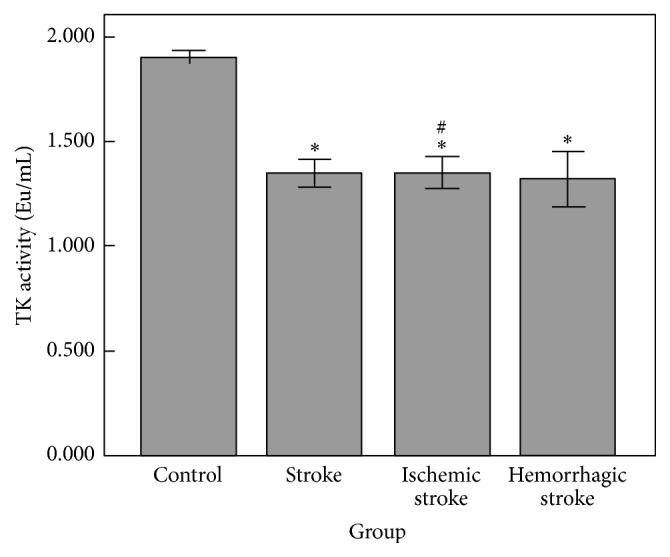
The mean TK activity (Eu/mL) in subjects grouped by type of stroke. ^*∗*^ *P* < 0.001 versus control, ^#^ *P* = 0.902 versus hemorrhagic stroke.

Significant differences in TK activity were not observed between ischemic stroke patients and hemorrhagic stroke patients (*P* = 0.962). Further study by the type of stroke showed that both ischemic stroke (1.582 ± 0.601 versus 1.934 ± 0.284 Eu/mL, *P* < 0.001) and hemorrhagic stroke patients (1.585 ± 0.751 versus 1.934 ± 0.284 Eu/mL, *P* < 0.001; Figure 3) had lower TK activity levels than controls.

### 3.3. Association of TK Activity with Stroke

The TK activity values were divided into 4 levels by quartiles as follows: quartile 1 (<1.600 Eu/mL), quartile 2 (1.600 to 1.936 Eu/mL), quartile 3 (1.936 to 2.052 Eu/mL), and quartile 4 (>2.052 Eu/mL). Univariate analysis showed no significant association between TK activity and traditional cardiovascular risk factors such as gender, age, body weight, BMI, SBP, DBP, HDL, TG, TC, history of hypertension, diabetes mellitus, hyperlipidemia, and smoking habits in controls grouped by quartiles, except for a univariate correlation with hyperlipidemia (*P* = 0.027) (Table 2).

TK activity level showed a strong negative association with risk of first-ever stroke in unconditional logistic regression analysis (OR, 0.47; 95% CI, 0.38–0.58; *P* < 0.001), when TK activity was used as a continuous variable after adjusting for traditional risk factors. Compared with quartile 1 of TK activity, the adjusted ORs of total stroke were 0.14 (95% CI, 0.07–0.29), 0.02 (95% CI, 0.01–0.04), and 0.10 (95% CI, 0.05–0.20) in quartiles 2, 3, and 4, respectively. These outcomes indicate that higher TK activity is independently associated with a significantly decreased risk of ischemic and hemorrhagic stroke (Table 3).

There were nonsignificant differences in TK activity among stroke patients with and without traditional cardiovascular risk factors, with the exception of SBP (*r* = 0.164, *P* = 0.003) and DBP (*r* = 0.206, *P* < 0.001). Further analysis by multivariable forward stepwise linear regression analysis showed that TG (Bata = −0.115; *P* = 0.040) and SBP (Bata = 0.110; *P* = 0.049) enter the regression equation in patients with stroke.

### 3.4. TK Activity Predicted Follow-Up Outcomes

Among the 321 patients of first-ever stroke, a total of 105 patients (including 47 ischemic stroke patients and 58 hemorrhagic stroke patients) experienced stroke recurrence during a median follow-up of 5 years (range, 4–5.5 years). We used multivariate Cox proportional hazard models to estimate the association of TK activity in both ischemic and hemorrhagic stroke patients with the recurrence of stroke after adjusting for traditional risk factors. The result of this analysis showed a dose-response relationship between higher TK activity and a markedly lower risk of recurrent stroke (adjusted RR, 0.69; 95% CI, 0.57–0.84; *P* < 0.001). In an unadjusted model using the lowest quartile as a reference group, the second, third, and fourth quartiles were associated with decreased risk of total recurrent stroke (*P* < 0.05). After adjusting for traditional risk factors, those in the higher quartiles of TK activity continued to have decreased risk of recurrent stroke. The adjusted RRs of total recurrent stroke were 0.59 (95% CI, 0.35–0.99), 0.44 (95% CI, 0.25–0.78), and 0.33 (95% CI, 0.18–0.61) in quartiles 2, 3, and 4, respectively (Table 4).

**Figure 4:**
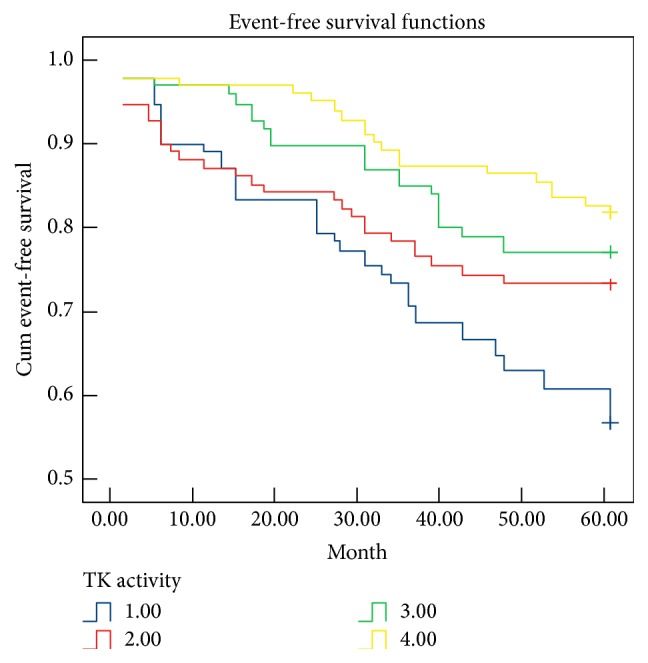
Kaplan-Meier survival curves based on TK activity. 1 = quartile 1; 2 = quartile 2; 3 = quartile 3; 4 = quartile 4.

Kaplan-Meier survival analysis was used to examine the association between TK activity, stroke recurrence, and event-free survival time. At 5-year follow-up, the rates of total stroke recurrence were 52.5%, 31.3%, 26.3%, and 21% for stroke patients in quartiles 1, 2, 3, and 4, respectively. The event-free survival time of patients in quartile 1 was 44.4 ± 2.3 months. Compared with patients in quartile 1, the event-free survival times of patients in quartiles 2, 3, and 4 were 48.1 ± 2.3 months (Log Rank test, *P* = 0.018), 52.1 ± 1.7 months (Log Rank test, *P* = 0.001), and 56 ± 1.3 months (Log Rank test, *P* < 0.001) (Figure 4). These data suggested that higher TK activity is predictive of fewer stroke recurrences and a longer event-free survival time.

## 4. Discussion

We examined TK activity in urine and evaluated the relationship between urinary TK activity and plasma TK levels for the first time. The results showed that urinary TK activity was correlated with plasma TK levels when the plasma TK level was below 0.200 mg/L. Patients with ischemic and hemorrhagic stroke exhibit markedly reduced TK activity, independent of other factors related to the risk of arterial disease. These results are consistent with our previous study, which showed that lower plasma TK levels were independently associated with the first occurrence of stroke [21]. These results further suggest that TK may be an independent endogenous protective factor against stroke in the Chinese population.

Previous studies have typically measured TK activity in urine using radioimmunoassay [27, 28]. Immunological and chemical technologies result in less radiocontamination and are comparatively safer. We therefore developed a novel assay to measure TK activity by utilizing its immunological characteristics [29] and the chemical characteristics of BAEE [23]. The results of this assay indicated that the TK activity is correlated with plasma TK levels. However, this association disappeared when the plasma TK level exceeded 0.200 mg/L.

TK has been demonstrated to elicit a broad spectrum of biological effects, such as reducing hypertension, cardiovascular and renal damage, restenosis, ischemic stroke, and skin wound injury. TK attenuates cardiovascular, renal, and brain damage by inhibiting oxidative stress, cell proliferation, apoptosis, inflammation, hypertrophy, atherosclerosis, and fibrosis and promoting angiogenesis and neurogenesis [14–18]. Transgenic and somatic gene transfer approaches have been used to achieve a continuous supply of TK and have shown that TK significantly reduces aortic thickening and the stroke-induced death rate in Dahl salt-sensitive rats [30]. TK provides neuroprotection against cerebral ischemia injury by enhancing glial cell survival and migration and inhibiting apoptosis through the suppression of oxidative stress and activation of the Akt-Bcl-2 signaling pathway [31].

In our previous study, higher plasma TK levels (below 0.200 mg/L) predicted a lower risk of stroke recurrence and a longer stroke-free interval over the course of a 5-year follow-up study. However, this association did not exist when the plasma TK level was over 0.200 mg/L [21]. In this report, subjects experiencing a subsequent stroke had significantly lower TK activity than first-ever stroke patients who had not experienced a recurrence. Our data showed that TK activity is negatively associated in a dose-dependent manner with overall stroke recurrence. These results might have several explanations. First, TK may both directly and indirectly activate the kinin B2 receptor and trigger an array of biological effects by increasing nitric oxide (NO)/cyclic 3′, 5′-guanosine monophosphate and cyclic adenosine monophosphate (cAMP) levels, thus reducing nicotinamide adenine dinucleotide phosphate oxidase activity and proinflammatory cytokine levels and activating the intracellular PI3 kinase/Akt and MAP kinase pathways, thereby protecting against atherosclerosis and cerebral injuries and preventing initial or recurrent stroke [32]. Second, traditional risk factors for stroke might impact plasma TK levels or TK activity. However, our previous study evaluated the association between TK level and activity in control subjects and found no significant statistical correlations by multivariate stepwise linear regression analysis, except for those with lipid metabolism. Cox proportional hazard models showed that plasma TK levels below 0.200 mg/L were independently and negatively associated with event and recurrent total stroke in a dose-dependent manner, regardless of whether adjustments were made for traditional stroke risk factors [21]. Therefore, these risk factors did not significantly impact plasma TK levels. In addition, an effect of these risk factors on TK activity was not observed. Third, TK protected humans from initial or recurrent stroke by activating endothelial NO synthase and increasing NO production, which triggers a broad spectrum of biological effects, including vasodilatation; smooth muscle contraction and relaxation; inhibition of apoptosis, atherosclerosis, inflammation, hypertrophy, and fibrosis; protection against ischemia/reperfusion damage; and promotion of angiogenesis and neurogenesis [14, 15]. Fourth, when plasma TK levels were above 0.200 mg/L, there was no association between plasma TK level and urinary TK activity, and there was no relationship between plasma TK level and the risk of stroke recurrence and stroke-free interval. Therefore, we suspected that plasma TK levels above 0.200 mg/L might inactivate the kinin B2 receptor and then suppress TK activity in urine. Further prospective and basic studies are needed to determine why plasma TK levels above 0.200 mg/L were not associated with urinary TK activity or the risk of stroke recurrence. Therefore, urinary TK activity may act as a protective factor against initial and recurrent stroke, and urinary TK activity may be a stronger predictor than plasma TK level of the recurrence of both ischemic and hemorrhagic strokes. Fifth, elevated TK may directly inhibit the proliferation, migration, and phenotype transformation of vascular smooth muscle cells by Rho/ROCK signaling pathway and protect human from stroke [33, 34]. In addition, DNA methylation, a powerful epigenetic regulator of endothelial transcription, plays an important role in the process of atherosclerosis [35, 36]. We, therefore, suspect that the methylation of KLK1 promoter region may affect TK activity and the occurrence of stroke. Therefore, studies on DNA methylation of the kallikrein-kinin genes will enhance the understanding of their role in stroke protection and provide insights into the predictor of stroke recurrent.

There are several limitations in the current study that should be addressed. First, both plasma TK levels and urinary TK activity were measured after stroke; therefore, we could not create a causal connection between stroke and reduced plasma TK level or urinary TK activity, nor could we determine whether changes in TK level and TK activity are predictors or consequences of first-ever stroke. Second, even though we adjusted for traditional risk factors in the multivariate stepwise linear regression analysis, the possibility of residual confounding elements remains. Third, the influence of diet on TK activity cannot be ruled out. Fourth, a causal link between reduced TK activity and recurrent stroke is biologically plausible and is supported by temporal and long-term associations and a dose-dependent correlation between urinary TK activity and long-term prognosis for stroke patients. Compared with plasma TK levels, urinary TK activities may be a stronger predictor of recurrent stroke. These issues can only be addressed through additional prospective studies of the association between TK activity and first-ever stroke. Additional studies could elucidate why stroke patients exhibit lower urinary TK activity.

## 5. Conclusions

This work, combined with our previous study, suggests that both the plasma TK level and urinary TK activity have a significant inverse relationship with first-ever stroke and stroke recurrence in the Chinese population. Furthermore, urinary TK activity may be a stronger predictor of recurrent stroke than plasma TK level. Large prospective human studies, as well as cellular and animal research, are needed to establish causality between stroke and TK activity.

## Figures and Tables

**Table 1:** General characteristics of stroke patients and controls.

Variable	Stroke patients	Controls	*P* value
*N* (male %)	321 (65)	323 (34)	0.121
Age, y	62.0 ± 10	62.6 ± 8	0.703
Hypertension, number (%)	203 (63)	136 (42)	0.034
Diabetes, number (%)	37 (12)	14 (4)	0.016
Hyperlipidemia, number (%)	60 (19)	91 (28)	0.003
Current smoker, number (%)	135 (42)	49 (15)	<0.001
DBP, mmHg	90 ± 15	81 ± 11	0.002
SBP, mmHg	147 ± 25	137 ± 21	0.887
Weight, kg	66 ± 12	62 ± 9	0.022
BMI, kg/m^2^	24 ± 4	24 ± 3	0.23
Total cholesterol, mmol/L	4.49 ± 1.01	5.23 ± 1.09	<0.001
Triglyceride, mmol/L	1.95 ± 1.0	1.65 ± 1.1	<0.001
HDL cholesterol, mmol/L	0.88 ± 0.3	1.29 ± 0.4	<0.001
TK activity, Eu/mL	1.583 ± 0.673	1.934 ± 0.284	<0.001

SBP: systolic blood pressure; DBP: diastolic blood pressure; BMI: body mass index; HDL: high-density lipoprotein cholesterol; TK: tissue kallikrein.

*P* values for the difference between stroke patients and controls, tested with independent samples *t*-test.

**Table 2:** The relationship between TK activity and cardiovascular disease risk factors in controls.

Variable	Quartile 1	Quartile 2	Quartile 3	Quartile 4	*P* value
TK activity, Eu/mL	<1.600	1.600–1.936	1.936–2.052	>2.052	
Number of participants	16	79	131	97	
Male sex, number (%)	10 (63)	25 (32)	44 (34)	30 (31)	0.091
Age, y	60 ± 7	63 ± 8	63 ± 8	62 ± 7	0.236
Hypertension, number (%)	4 (25)	34 (43)	52 (40)	45 (46)	0.390
Diabetes, number (%)	0 (0)	6 (1)	4 (0)	4 (0)	0.300
Hyperlipidemia, number (%)	0 (0)	19 (24)	38 (29)	34 (35)	0.027
Current smoker, number (%)	4 (25)	9 (11)	23 (18)	13 (13)	0.407
Weight, kg	63 ± 9	62 ± 10	61 ± 9	62 ± 9	0.392
BMI, kg/m^2^	24 ± 2	24 ± 3	23 ± 3	24 ± 3	0.555
SBP, mmHg	136 ± 6	138 ± 20	137 ± 21	136 ± 21	0.883
DBP, mmHg	85 ± 6	82 ± 11	81 ± 11	81 ± 11	0.481
Total cholesterol, mmol/L	4.89 ± 1.04	5.41 ± 0.91	5.13 ± 1.16	5.26 ± 1.13	0.192
Triglyceride, mmol/L	2.05 ± 1.28	1.62 ± 0.99	1.59 ± 1.25	1.69 ± 1.07	0.462
HDL, mmol/L	1.21 ± 0.33	1.30 ± 0.38	1.33 ± 0.44	1.24 ± 0.32	0.286

TK: tissue kallikrein; BMI: body mass index; SBP: systolic blood pressure; DBP: diastolic blood pressure; HDL: high-density lipoprotein cholesterol.

**Table 3:** Unconditional logistic regression analysis for stroke and TK activity.

Group measure	Quartile 1	Quartile 2	Quartile 3	Quartile 4
TK activity, Eu/mL	<1.600	1.600–1.936	1.936–2.052	>2.052
Total stroke	145	82	30	64
Unadjusted (OR)^†^	1	0.11 (0.06–0.21)	0.03 (0.013–0.05)	0.07 (0.04–0.12)
Adjusted (OR)^†^	1	0.14 (0.07–0.29)	0.02 (0.01–0.04)	0.10 (0.05–0.20)
Ischemic stroke	66	58	20	30
Unadjusted (OR)^†^	1	0.81 (0.52–1.27)	0.20 (0.11–0.37)	0.32 (0.19–0.52)
Adjusted (OR)^†^	1	1.12 (0.66–1.88)	0.30 (0.16–0.59)	0.55 (0.32–0.97)
Hemorrhagic stroke	79	24	10	34
Unadjusted (OR)^†^	1	0.18 (0.11–0.31)	0.07 (0.03–0.14)	0.26 (0.16–0.41)
Adjusted (OR)^†^	1	0.20 (0.10–0.38)	0.07 (0.03–0.16)	0.37 (0.21–0.68)

TK: tissue kallikrein; ORs: odds ratios.

^†^ORs (95% confidence interval). Adjusted ORs have been adjusted for age, sex, presence or absence of hypertension, diabetes, hyperlipidemia, smoking, weight, BMI, SBP, DBP, total cholesterol, triglyceride, and HDL.

**Table 4:** The association between TK activity and stroke recurrence.

Stroke recurrence	Quartile 1	Quartile 2	Quartile 3	Quartile 4
Total stroke, *n*	80	80	80	81
Events, number (%)	42 (52.5)	25 (31.3)	21 (26.3)	17 (21)
Unadjusted (RR)^†^	1	0.55 (0.34–0.91)	0.43 (0.25–0.72)	0.32 (0.18–0.54)
*P* value		0.019	0.001	<0.001
Adjusted (RR)^†^	1	0.59 (0.35–0.99)	0.44 (0.25–0.78)	0.33 (0.18–0.61)
*P* value		0.048	0.005	<0.001
Ischemic stroke	40	37	55	42
Events, number (%)	22 (55)	14 (37.8)	15 (27.3)	7 (16.7)
Unadjusted (RR)^†^	1	0.71 (0.36–1.39)	0.45 (0.23–0.87)	0.25 (0.11–0.58)
*P* value		0.321	0.018	0.001
Adjusted (RR)^†^	1	0.90 (0.43–1.89)	0.59 (0.28–1.22)	0.30 (0.12–0.75)
*P* value		0.779	0.155	0.01
Hemorrhagic stroke	40	43	25	39
Events, number (%)	20 (50)	11 (25.6)	6 (24)	10 (25.6)
Unadjusted (RR)^†^	1	0.44 (0.21–0.92)	0.39 (0.16–0.98)	0.39 (0.18–0.84)
*P* value		0.028	0.046	0.016
Adjusted (RR)^†^	1	0.27 (0.12–0.63)	0.16 (0.06–0.46)	0.17 (0.07–0.46)
*P* value		0.002	0.001	<0.001

TK activity in quartile 1 (<1.063 Eu/mL), quartile 2 (1.063–1.667 Eu/mL), quartile 3 (1.667–1.988 Eu/mL), and quartile 4 (>1.988 Eu/mL).

TK: tissue kallikrein; RRs: relative risks. ^†^RRs (95% confidence interval). Adjusted RRs have been adjusted for age, sex, presence or absence of hypertension, diabetes, hyperlipidemia, smoking, weight, BMI, SBP, DBP, total cholesterol, triglyceride, and HDL.
